# Usefulness of Hybrid PET/MRI in Clinical Evaluation of Head and Neck Cancer Patients

**DOI:** 10.3390/cancers12020511

**Published:** 2020-02-22

**Authors:** Natalia Samolyk-Kogaczewska, Ewa Sierko, Dorota Dziemianczyk-Pakiela, Klaudia Beata Nowaszewska, Malgorzata Lukasik, Joanna Reszec

**Affiliations:** 1 Department of Radiotherapy, Comprehensive Cancer Center, 15-027 Bialystok, Poland; natalia.samolyk@gmail.com; 2 Department of Oncology, Medical University of Bialystok, 15-027 Bialystok, Poland; 3 Department of Otolaryngology and Maxillofacial Surgery, Jedrzej Sniadecki Memorial Regional Hospital, 15-950 Bialystok, Poland; dyrka2@wp.pl; 4 Department of Maxillofacial and Plastic Surgery, Medical University of Bialystok, 15-276 Bialystok, Poland; beata.nowaszewska@wp.pl; 5 Department of Medical Pathology, Medical University of Bialystok, 15-089 Bialystok, Poland; mblukasik@gmail.com (M.L.); joannareszec@gmail.com (J.R.)

**Keywords:** PET/MRI, head and neck cancer, HPV, EBV, p16

## Abstract

(1) Background: The novel hybrid of positron emission tomography/magnetic resonance (PET/MR) examination has been introduced to clinical practice. The aim of our study was to evaluate PET/MR usefulness in preoperative staging of head and neck cancer (HNC) patients (pts); (2) Methods: Thirty eight pts underwent both computed tomography (CT) and PET/MR examination, of whom 21 pts underwent surgical treatment as first-line therapy and were further included in the present study. Postsurgical tissue material was subjected to routine histopathological (HP) examination with additional evaluation of p16, human papillomavirus (HPV), Epstein-Barr virus (EBV) and Ki67 status. Agreement of clinical and pathological T staging, sensitivity, specificity, positive predictive value (PPV), negative predictive value (NPV) of CT and PET/MR in metastatic lymph nodes detection were defined. The verification of dependences between standardized uptake value (SUV value), tumor geometrical parameters, number of metastatic lymph nodes in PET/MR and CT, biochemical parameters, Ki67 index, p16, HPV and EBV status was made with statistical analysis of obtained results; (3) Results: PET/MR is characterized by better agreement in T staging, higher specificity, sensitivity, PPV and NPV of lymph nodes evaluation than CT imaging. Significant correlations were observed between SUVmax and maximal tumor diameter from PET/MR, between SUVmean and CT tumor volume, PET/MR tumor volume, maximal tumor diameter assessed in PET/MR. Other correlations were weak and insignificant; (4) Conclusions: Hybrid PET/MR imaging is useful in preoperative staging of HNC. Further studies are needed.

## 1. Introduction

Head and neck cancers (HNC) are the sixth most common cancers worldwide. The vast majority (more than 90%) are squamous cell carcinomas, so that the term HNC is often used to describe all carcinomas arising from the epithelium lining the sinonasal tract, oral cavity, pharynx and larynx and showing microscopic evidence of squamous differentiation. Approximately two-thirds of patients with HNC present with locally advanced disease at the primary site and/or spread to regional lymph nodes. The 5-year survival rate of HNC patients ranges between 40% and 70%, depending on the primary site and stage [1,2]. Positron emission tomography (PET) with fluorine-18 labelled fluorodeoxyglucose (18F-FDG) as a radiotracer is an established procedure in head and neck squamous cell carcinoma (HNSCC) diagnosis [2]. It is vitally important to the characterization of local, regional and distant disease [3]. PET supports the initial staging of difficult cases, where other imaging methods produce equivocal results. It is useful in the detection of occult primary tumors and synchronous primary cancers. Furthermore, PET aids in assessing tumor response to therapy and helps in detecting cancer recurrence and metastasis during the follow-up period [3,4,5].

Contrary to anatomical imaging, 18F-FDG-PET takes into account biological differences in metabolism and the radiopharmacological uptake between the tumor and the surrounding normal tissue [6]. PET images are characterized by high contrast but low spatial resolution, which creates a need for combining PET with morphological imaging methods such as cone beam computed tomography (CBCT). A novel hybrid imaging technology, Positron Emission Tomography-Magnetic Resonance Imaging (PET/MRI), has been introduced to clinical practice [7]. The integrated PET/MRI technique combines the benefits of both methods such as superior soft tissue contrast, multiplanar image acquisition and functional imaging capability [8]. 

A suspicion of head and neck cancer (HNC) is predominantly based on the identification of a primary tumor or the presence of palpable metastatic lymph nodes in the head and neck region during a clinical examination [9]. Accurate, pre-treatment staging is crucial in newly diagnosed HNSCC patients since it enables clinicians to determine patient prognosis and develop an optimal treatment strategy [5]. Precision imaging methods play an integral role in this process [4]. Furthermore, proper imaging is vital to the accurate delineation of target volumes in radiotherapy (RT) planning, particularly when highly conformal RT techniques such as intensity modulated radiotherapy (IMRT) or volumetric arc therapy (VMAT) are utilized [9]. Initial reports have revealed that hybrid 18F-FDG PET/MRI offers higher sensitivity and specificity in the detection of HNC when compared to PET, CT and MRI used separately [10,11]. Moreover, PET/MRI has a higher correlation coefficient with pathological tumor size in comparison to contrast-enhanced CT (CECT), MR and PET/CT [10]. PET/MRI outperformed PET/CT by providing greater tumor conspicuity and higher sensitivity in determining the perineural spread in primary HNSCC. One study has demonstrated PET/MRI sensitivity to be 85% and specificity 92% in the detection of metastatic lymph nodes [3].

In HNSCC patient population, a group of younger, healthier individuals with limited or no tobacco exposure can be distinguished [12]. Prognosis in such cases is excellent as these patients are highly responsive to treatment [13]. Human papillomavirus (HPV)-related oropharyngeal cancers (OPC) are a frequent finding in this group of patients. Presence of HPV is an important prognostic factor, which is reflected in the 8th edition of the American Joint Committee on Cancer (AJCC) TNM (Tumor Nodes Metastases classification) staging system [13,14]. There is a limited number of studies on the relationship between PET imaging and HPV status in HNSCC [3,15,16,17]. Furthermore, very few studies concern the utility of PET/MRI in the characterization of HPV-related HNC [8,18]. According to our knowledge, there are no literature reports documenting the correlation between Epstein-Barr virus (EBV) status and PET/MRI features. Therefore, the aim of our study was to evaluate the correlation between PET/MR and CT parameters, biochemical parameters, Ki67 index, p16, HPV and EBV status as well as usefulness of PET/MRI in the preoperative staging of HNC patients. 

## 2. Results

This section may be divided by subheadings. It should provide a concise and precise description of the experimental results, their interpretation as well as the experimental conclusions that can be drawn.

### 2.1. Accuracy of T staging

Agreement between T stages determined with CT, PET/MRI and histopathological (HP) examination, which is the clinical gold-standard technique used for cancer diagnosis, was evaluated. The T stage determined with CT was compatible with the HP stage in 56% of cases. T overstaging was observed in 11% of cases, downstaging in 33% of patients. PET/MRI results corresponded to HP staging in 67% of cases, were overstaged in 11%, downstaged in 22%.

### 2.2. Specificity, Sensitivity, Positive Predictive Value (PPV) and Negative Predictive Value (NPV) of Lymph Node Evaluation in CT and PET/MRI

The total number of detected lymph nodes in the HP examination of postoperative tissue material was 475, out of which 31 were metastatic. Twenty seven lymph nodes were described as malignant in CT scans and 24 in PET/MRI scans. Based on the CT and PET/MRI scans, lymph nodes of the neck were assigned into appropriate levels [19]. Results of the HP were referenced values. In regard to lymph node detection, PET/MRI sensitivity was 55%, specificity—98%, PPV—71%, NPV—97% and test reliability was 95%. Computed tomography sensitivity was 47%, specificity—97%, PPV—53%, NPV—96% and test reliability—94%.

### 2.3. Correlations between Clinical, Morphological and Metabolic Parameters

#### 2.3.1. SUV Values in Relation to Tumor Geometrical Parameters, Biochemical Parameters and Ki67 Index

**Figure 1:**
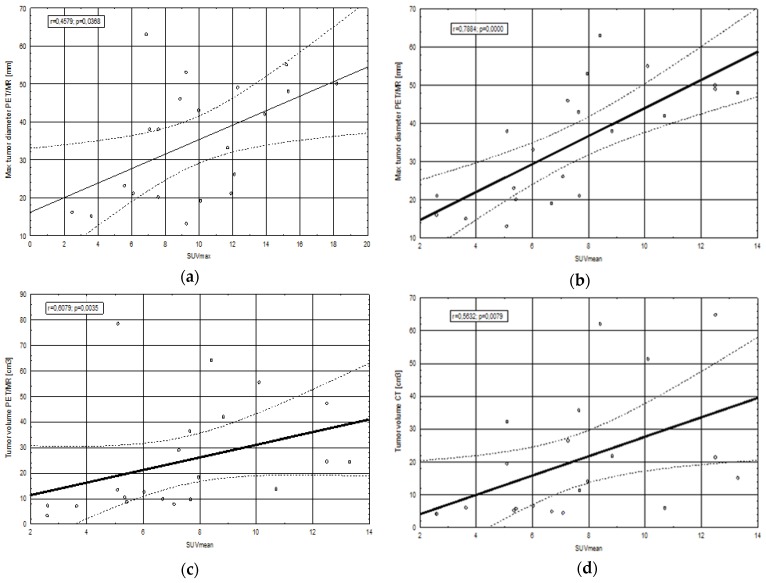
Statistically significant correlation between standardized uptake value (SUV) values and maximal tumor diameter (**a**,**b**) and tumor volume (**c**,**d**) obtained from positron emission tomography/magnetic resonance (PET/MR) or computed tomography (CT). Abbreviations: SUVmax—maximal standardized uptake value, SUVmean—mean standardized uptake value, r—correlation coefficient from Spearman’s correlation test, *p*—statistical significance value.

A high, statistically significant correlation between standardized uptake value (SUV)max and maximal tumor diameter determined with PET/MRI (r = 0.457, *p* = 0.036) (Figure 1) was revealed in comparison to the measurements obtained from CT images (r = 0.025, *p* = 0.913). On the other hand, poor and statistically insignificant correlations were observed between SUVmax values and tumor volume determined with PET/MRI (r = 0.309, *p* = 0.172), tumor volume determined with CT (r = 0.289, *p* = 0.203), Ki67 index (r = 0.04, *p* = 0.862), CRP concentration (r = 0.069, *p* = 0.764) and glucose levels (r = 0.308, *p* = 0.173). 

A significant relationship was observed between SUVmean and CT tumor volume (r = 0.563, *p* = 0.007), PET/MRI tumor volume (r = 0.607, *p* = 0.003) and maximal tumor diameter determined with PET/MRI (r = 0.788, *p* = 0.00002) (Figure 1). Contrary to the aforementioned findings, correlations between SUVmean and maximal tumor diameter in CT (r = 0.165, *p* = 0.474), Ki67 index (r = 0.08, *p* = 0.728), CRP concentration (r = 0.138, *p* = 0.549) and glucose levels (r = 0.253, *p* = 0.268) were weak and insignificant. 

#### 2.3.2. Presence of p16, HPV and EBV in Relation to SUV Values, Tumor Geometrical Parameters 

The average value of SUVmax in p16-positive patients was lower than in p16-negative patients. Despite substantial discrepancies in the obtained results, the difference between the p16-positive and the p16-negative groups was insignificant (*p* = 0.37). Maximal SUV was not significantly correlated with HPV and EBV status, and neither was maximal tumor diameter determined with CT or PET/MRI correlated with the presence of p16, HPV, EBV. 

The average values of SUVmean in p16-, HPV- and EBV-positive patients were close to the values in p16-, HPV- and EBV-negative groups.

#### 2.3.3. Number of Metastatic Lymph Nodes in Relation to SUV Values, Tumor Geometrical Parameters, Ki67 Index and Presence of p16, HPV, EBV

The correlation between CT-based tumor volume and the number of metastatic lymph nodes determined with CT was statistically significant (*p* = 0.006). The highest tumor volumes were found in the group of patients with 1 metastatic lymph node and the lowest in those without metastatic lymph nodes (*p* = 0.01). The number of metastatic lymph nodes determined with CT or PET/MRI and SUV values, PET/MRI-based tumor volume, maximal tumor diameter based on CT or PET/MR imaging, Ki67 index were insignificant. 

No significant correlations were observed between the presence of p16 (*p* = 0.431, df = 2, Chi2 = 1.68), HPV (*p* = 0.623 df = 2, Chi2 = 0.946), EBV (*p* = 0.714 df = 2, Chi2 = 0.674) and the number of metastatic lymph nodes detected with CT, as well as between the positive status of p16 (*p* = 0.24 df = 2, Chi2 = 2.854), HPV (*p* = 0.139 df = 2, Chi2 = 3.94), EBV (*p* = 0.414 df = 2, Chi2 = 1.763) and the number of metastatic lymph nodes detected with PET/MRI.

## 3. Discussion

Hybrid PET/MRI imaging can play an important role in the initial staging of HNC patients. In our study, we compared the efficacy of PET/MRI with the routinely used CT scanning and HP examination, which is the clinical gold-standard technique used for cancer diagnosis, in primary tumor staging (T features) according to the AJCC TNM classification. Our results demonstrated that T staging based on PET/MRI images was compatible with HP outcomes in a higher number of patients (67%) in comparison with T staging based on CT images alone (56%). Similar results were obtained by other authors - T staging performed with PET/MRI was accurate in 75% of HNC cases compared to 59% with PET/CT and 50% with MRI alone [20]. Chan et al. demonstrated that greater accuracy in defining the extent of primary nasopharyngeal tumors was achieved with PET/MRI in comparison to PET/CT [21]. PET/MRI imaging offers more precision in depicting perineural infiltration, intracranial or inferior orbital fissure involvement. It should be mentioned that our study did not include nasopharyngeal cancer (NPC) patients. Other authors have demonstrated similar results: fused PET and MR imaging delivered more accurate T staging of HNC patients (74%) compared with PET/CT (63%) [22]. Tsujikawa et al. demonstrated that FDG PET/MRI with its greater soft tissue contrast, multiplanar image acquisition and functional imaging capability is useful in the evaluation of stages of oral cavity cancers and the assessment of oropharyngeal cancer patients based on the new AJCC TNM staging system [8]. 

Regional metastasis to the lymph nodes is one of the most important predictors of poor prognosis in HNC [23]. Based on the results of CT and PET/MRI scans, we calculated the sensitivity, specificity, PPV and NPV of both diagnostic tests. Positive predictive value expresses the probability that a positive diagnostic test result is reliable. Similarly, NPV determines the probability that a negative test result is reliable [24]. Our study revealed relatively low sensitivity of PET/MRI in metastatic lymph node detection. This could be associated with the fact that detecting small nodes with micrometastases remains a challenge for currently available diagnostic modalities, including PET/MRI [2]. Metastases can be present in unenlarged lymph nodes and not all enlarged nodes are malignant [25]. Another explanation for such results may be limited radiological expertise in interpreting PET/MRI images as this is a novel hybrid imaging technique. Our study has found that specificity, NPV and test reliability of PET/MR is marginally better than in CT results. On the other hand, PET/MR has 8% higher sensitivity and 18% higher PPV compared with CT. In a different study, the sensitivity, specificity and accuracy of PET/MRI-based evaluation of regional node status achieved a 99% rate each [21]. Based on lymph node levels, Schaarschmidt et al. demonstrated sensitivity, specificity, NPV and PPV of 81%, 99%, 98% and 89%, respectively, for PET/MRI [20]. On the other hand, Platzek et al. found that PET/MRI is capable of detecting 64% more metastatic lymph nodes, partly due to the fact that PET/MRI scans were performed following PET/CT scans without the additional FDG injection and the time interval between the FDG injection and PET/MR was longer, which contributed to an increase in FDG uptake by the lymph nodes [26]. Another study has highlighted a better diagnostic value of PET/MRI staging in primary tumors compared with cervical node classification in head and neck malignancies with 94% of primary tumors and 82% of regional nodes correctly classified in comparison to histopathology results [27].

Positron emission tomography with FDG as a radiotracer reflects the metabolic activity of tumor tissue which is expressed in SUV values [28]. Our study revealed a stronger correlation between SUVmean and tumor geometry, such as volume and maximal diameter, than between SUVmax and tumor geometry. PET complements anatomical imaging modalities, particularly in tumor volume determination. Therefore, our study demonstrated stronger correlations between SUV values and PET/MRI than between those values and CT [29]. Tumor volume and SUV values are considered prognostic factors—increased values of both are associated with a higher risk of death [30,31]. Biologically, larger tumors have higher metabolic activity and therefore, higher SUV values indicate a worse prognosis [32]. PET/MRI has an advantage over CT as it enables the measurements of tumor volume and SUV values to be obtained during one examination. 

Poor and statistically insignificant correlations were found between SUVmax, SUVmean and KI67, CRP, glycemic parameters in our study. The authors of a meta-analysis concerning correlations between FDG uptake and Ki67 reported significant heterogeneity in HNC cases [33]. Furthermore, pretreatment SUVmax was significantly higher in patients with enhanced Ki67 expression in comparison to those with low Ki67 expression, but the study concerned diffuse large B-cell lymphoma [34]. Surov et al. reported that SUVmax was moderately correlated with Ki67 expression and therefore cannot be used as a surrogate marker for tumor proliferation [35]. A Chinese study on a group of lymphoma patients showed that post-treatment SUVmax values were significantly correlated with post-treatment CRP values [36]. By contrast, a previously mentioned study demonstrated that baseline SUVmax was weakly correlated with CRP (r = 0.215, *p* = 0.011) [34].

A recent meta-analysis investigating the effect of blood glucose levels on SUV values assessed with FDG-PET scans revealed positive correlations between glucose levels and SUVmax and SUVmean values in normal tissue such as brain, muscle, liver, but no significant correlations were found between blood glucose levels and SUVmax or SUVmean values in primary malignant tumors [37]. No evidence of a linear relationship between glycaemia and SUV has been reported by other authors [38]. However, it should be emphasized that high plasma glucose levels decrease FDG uptake, which might affect SUV values [39].

In the present study, differences between SUVmax or SUVmean and HPV or p16 presence were insignificant. Comparable results have been obtained in other studies, where no differences in SUVmax values between p16- and HPV-positive, and p16- and HPV-negative patients were reported [40,41,42]. By contrast, Schouten et al. demonstrated that SUVmax in HPV-positive tumors was significantly lower than in HPV-negative tumors [43]. This finding is in agreement with a study by Surov et al. which revealed that p16-negative tumors showed significantly higher SUVmax and SUVmean values in comparison to p16-positive carcinomas [44]. 

In our study, no correlation between SUV values and EBV infection status was observed. Literature data confirms our results [45,46].

Similarly, in the present study p16, HPV, EBV did not have a significant impact on the results of CT- or PET/MRI-determined tumor volume and maximal tumor diameter. However, according to the literature, in HNC patients, p16/HPV-positive tumors are usually smaller than p16/HPV-negative tumors [44,47]. Some studies have demonstrated that EBV status and primary tumor volume can be utilized in the prognostic stratification of NPC patients [48,49]. On the other hand, data confirming correlations between primary tumor volume and EBV-positivity is limited [50,51]. 

Maximal tumor diameter is an important primary tumor feature which is reflected in the TNM classification of HNC (e.g., T1 denotes a tumor size 2 cm or less in its largest dimension) [13]. There are a few studies demonstrating the prognostic value of maximal tumor diameter in OPC [52] and NPC [53], but there are no published papers concerning the influence of p16-, HPV- or EBV-positivity on maximal tumor diameter determined with CT or PET/MRI.

According to our knowledge, there are no literature reports examining the relationship between PET/MRI-based tumor volume and the number of metastatic lymph nodes in HNC patients. Our study did not find any correlations between tumor features obtained from PET/MRI scans and the number of metastatic lymph nodes. By contrast, correlations between tumor volume and the number of metastatic lymph nodes based on CT images were significant. The lowest tumor volumes determined with CT correlated with no metastases in the lymph nodes. This is normally the case in early stage HNC patients. We therefore expected to find significant correlations between the highest tumor volumes determined with CT and multiple metastatic lymph nodes. However, the highest CT-based tumor volumes were observed in patients with 1 metastatic lymph node. This can be explained by the highly inflammatory nature of HNSSC, particularly at advanced stages of the disease [54]. Large tumors investigated in the present study probably consisted not only of neoplastic tissue but also contained peritumoral inflammatory infiltrate. On the other hand, HNC has a high propensity to metastasize through the lymphatic pathway [55]. This could explain the occurrence of multiple metastases in the lymph nodes independently of tumor volumes presented in our study. Research concerning lung adenocarcinoma has demonstrated positive correlations between tumor size and the number of metastatic lymph nodes [56]. By contrast, Miller et al. reported that tumor volume in cervical cancer patients did not correlate with the presence of lymph node metastases [57]. 

In the present study, the correlation between the number of metastatic lymph nodes determined with PET/MRI and primary tumor SUV values was insignificant. Only few papers have explored this issue but the results obtained were contradictory. Some authors report that in lung cancer patients no statistically significant differences in SUVmax and SUVmean values between individuals with occult lymph nodes and those without have been found [58]. A different study has demonstrated that SUVmax and SUVmean are significantly associated with lymph node involvement in oesophageal cancer patients [59].

Correlations between the presence of HPV, p16 and EBV, and the number of metastatic lymph nodes determined with PET/MRI were insignificant in our study. Other authors have obtained similar results—HPV-positive patients were heterogenous in terms of nodal stages [60]. By contrast, in TROG 02.02 phase III trial, p16-positive patients were included in a more advanced nodal category in comparison with the p16-negative group [47]. The vast majority of NPC is linked to Epstein-Barr virus infection [61,62]. A lack of relationship between EBV status and the number of lymph nodes detected with PET/MRI is most probably associated with the inclusion of oropharyngeal and oral cavity cancer patients in our study.

Based on our results, further studies should include a larger number of HNC pts. 

## 4. Materials and Methods 

**Figure 2:**
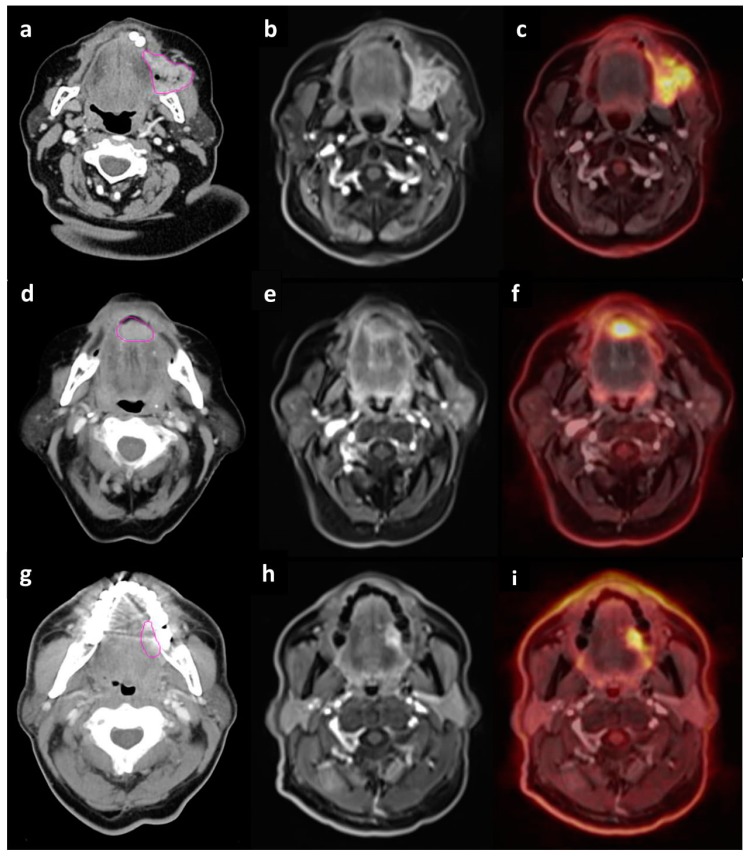
Contrast enhancement computed tomography (CT) (**a**,**d**,**g**), T1-weighted volumetric interpolated breath hold examination (VIBE) magnetic resonance (MR) (**b**,**e**,**h**) and positron emission tomography/MR (PET/MR) (**c**,**f**,**i**) imaging of head and neck cancer patients. (**a**,**b**,**c**)—patient with squamous cell carcinoma of the buccal mucosa in clinical stage T3N2b; (**d**,**e**,**f**)—patient with squamous cell carcinoma of the floor of the mouth in clinical stage T1N0; (**g**,**h**,**i**)—patient with squamous cell carcinoma of the oral tongue in clinical stage T2N0.

Thirty eight HNSCC patients underwent both CT and PET/MRI examinations. (Figure 2) Based on the CT and PET/MRI results, 21 patients underwent surgical treatment as first-line therapy and this group was subsequently included in the present study. The remaining patients received radiochemotherapy or palliative RT. Study inclusion criteria were as follows: age over 18 years old, informed consent for study participation, no unmanaged systemic diseases, no hypersensitivity or previous allergic reaction to intravenous iodine contrast or FDG, glucose blood level below 160mg/dL, no metal elements in the body (cardiac pacemakers, metal surgical stitches or clips, intrauterine contraceptive devices, metal shavings in the eyeball, cochlear implants, etc.). Clinical characteristics of the group are presented in Table 1. All pts had squamous cell carcinoma and in about 87% of cases histology grade score (G) was G2. The median age of study participants was 60 years (range of 36–74 years). Twelve of them were female, nine were male. Hypertension and diabetes were the most common comorbidities. In terms of laboratory parameters (Complete Blood Count, renal and liver function parameters, electrolyte blood concentration, thyroid hormones level), the group of patients was homogenous. Discrepancies were observed in C reactive protein (CRP) levels in the blood and its values ranged from 0.3 mg/mL to 120.5 mg/mL.

Prior to surgical treatment, contrast-enhanced (Ultravist 300, 1 mL/kg) CT was routinely performed in all study participants on the 64-detector row CT scanner (Aquilion CX, Canon Medical Systems Corporation, Otawara, Japan). Axial images were obtained with 3 mm slice thickness from the top of the cranium to manubrium sterni. The parameters included a 120 kV tube voltage, tube current 200 mA, 0.5 s rotation time, matrix size, 512 × 512 and a field of view of 250 mm. CT scans were interpreted by a radiologist experienced in the interpretation of head and neck region CT images. 

All study participants also underwent PET/MRI scans on the 3 Tesla Siemens Biograph mMR scanner (Siemens Healthcare GmbH, Erlangen, Germany) after an average of 9 days (range 1 to 20 days) from the CT scans. The examination consisted of whole-body, low resolution MRI imaging, followed by a PET scan and a diagnostic MRI scan (T1- and T2-weighted sequences and contrast-enhanced sequences) of the head and neck region. MRI attenuation images were acquired using the Dixon approach with a coronal 2-point 3D T1-weighted volumetric interpolated breath hold examination (VIBE) (3.12 mm slice thickness, 20% interslice gap, integrated parallel acquisition technique factor 2, acquisition time 19s, 192 × 121 matrix, 500mm × 328 mm field of view (FOV), repetition time 3.6ms, echo time 1.23 and 2.46ms, scanning time 4 minutes 10 seconds). PET/MRI scans were obtained simultaneously with the patient remaining in the same position without specific head and neck immobilization (Orfit mask). All patients fasted for at least 6 hours prior to the test. Diagnostic scans were performed 1 hour after the intravenous administration of 18F-FDG (4 MBq/kg, range of 203–417 MBq 18F-FDG per patient). PET/MRI images were evaluated by both a radiologist and nuclear medicine specialist. Maximal and mean SUV of the primary volume were measured. Mean SUV was defined as the average SUV value in the voxel that showed ≥ 40% of SUVmax [63].

Surgical treatment was performed an average of 19 days (range of 9 to 28 days) after the CT scans and an average of 13 days (range of 2 to 27 days) after the PET/MRI scans. 

Tissue material obtained during surgery was subjected to routine histopathological examination (HP). Additionally, the HP protocol included the immunohistochemical evaluation of p16 protein expression as a surrogate marker for HPV infection, the latent membrane protein 1 (LMP1) expression as a surrogate marker for EVB as well as the detection of Ki67 protein indicating the proliferation rate of cancer cells. The immunohistochemical evaluation of p16 protein expression was conducted using a method described elsewhere [64,65]. Slides were considered positive for p16 protein if the specimen showed continuous staining of at least 50% of tumor cells. For the immunohistochemical detection (Dako, Poland) of EBV presence, antibodies against LMP1―monoclonal mouse anti-LMP protein (clone CS.1-4, Dako, Poland) were used. The expression of Ki67 protein was evaluated with an immunohistochemical method described in the literature [66]. Nuclear accumulation of Ki67 protein in neoplastic cells was assessed semiquantitatively and defined as a percentage of reaction-positive cells.

The presence of HPV genetic material was examined using the PCR method. A positive result (HPV presence in the analyzed material) was determined when at least one of the bands associated with a different HPV subtype was present on the strip along with the developing control line and the positive control. A negative result (no HPV presence) was determined when no bands associated with HPV were developed while the developing control line and the positive control band were both present.

Clinical and pathological stages of the disease were determined according to the 8th ed. of AJCC TNM classification [8]. Based on imaging tests, only round-shaped lymph nodes or nodes exceeding 10mm in the smallest transverse dimension with contrast enhancement on CT or MRI and/or increased 18F-FDG uptake in PET scans were taken into consideration. The results obtained from CECT, PET/MRI and HP were compared. HP results were referenced. 

Agreement between CT- and PET/MRI-based T staging, sensitivity, specificity, PPV, NPV in metastatic lymph node detection were defined. Sensitivity was the proportion of patients with positive diagnostic test results (CT or PET/MRI scanning) who were correctly diagnosed in relation to referenced HP results. Specificity was the proportion of patients with negative imaging test results who were correctly diagnosed [67]. Positive predictive value is a proportion of true positive results among all positive findings (true positives and false positives). Similarly, negative predictive value is a proportion of true negative results among all negative results (true negatives and false negatives) [22]. 

Dependences between SUVmax and SUVmean values, the size and maximal diameter of the primary tumor, the number of metastatic lymph nodes detected with PET/MRI, HPV and EBV status, Ki67 index, glucose level and CRP concentration status were verified in the statistical analysis of the obtained results (Table 2). Considering the number of lymph nodes, study participants were divided into 3 groups: those without metastatic lymph nodes, those with 1 metastatic lymph node and those with 2 or more metastatic lymph nodes. The division was based on the 8th ed. of AJCC TNM classification of HNC, where N0 means no lymph node metastasis, N1–metastasis in single small lymph node and N2–among others, metastasis in multiple lymph nodes.

Statistical analysis was performed using Microsoft Excel (version 2019, Microsoft Corporation, Washington, DC, USA) and Statistica 10 (Statsoft Inc., Tulsa, OK, USA). The level of statistical significance was established at *p* < 0.05. The Spearman’s correlation test was used to analyze correlations between SUVmax/SUVmean values and maximal tumor diameter determined with CT or PET/MRI, CT or PET/MRI tumor volume, Ki67 index, CRP concentration, glucose level. The correlations between SUVmax, SUVmean, maximal tumor diameter determined with CT or PET/MRI and p16, HPV, EBV presence were analyzed using the t test. Correlation analysis between CT or PET/MRI tumor volume, Ki67 index, CRP concentration and p16, HPV, EBV status was performed with the Mann–Whitney U test. Correlations between the number of metastatic lymph nodes determined with CT or PET/MRI and features such as SUV values, maximal tumor diameter and tumor volume determined with CT or PET/MRI, KI67 index were established with the Kruskal–Wallis test. The Ch2 test was used to analyze the relationship between the number of metastatic lymph nodes determined with CT or PET/MRI and the positivity of p16, HPV and EBV. 

## 5. Conclusions

Hybrid PET/MRI imaging is useful in the preoperative staging of HNC patients. Compared with CT scanning, the outcomes obtained using this imaging modality are more consistent with histopathology results, the gold-standard technique in cancer diagnosis, in regard to T staging. This novel imaging tool also offers higher specificity, sensitivity, PPV and NPV of lymph node evaluation in comparison with CT scanning. Statistically important correlations between SUVmax and maximal tumor diameter as well as between SUV mean and tumor volume and maximal tumor diameter evaluated with PET/MRI were revealed in the present study, which suggests that these parameters may be useful in clinical practice. The presence of p16, HPV, EBV did not correlate with SUV values, geometrical parameters of the tumor or the number of metastatic lymph nodes. Further studies on a larger group of patients are required.

## Figures and Tables

**Table 1:** Characterization of patient with head and neck squamous cell carcinoma (SSC) according to localization of the primary tumor, Ki67 expression, clinical stage (TNM classification, AJCC, ed. 8, 2017) based on postoperative histopathological (HP) examination, status of p16 protein, human papilloma virus (HPV) and Epstein-Barr virus (EBV) positivity, smoking status and biopsy performed before positron emission tomography/magnetic resonance (PET/MR) imaging.

No. of pts.	Localization of Primary Tumor	TNM Stage	Ki67 Index [%]	p16 Status	HPV Status	EBV Status	Smoking	Biopsy before PET/MR (days)
1	OT	T2 N1 M0	30	0	0	0	1	28
2	BM	T2 N1 M0	30	0	0	0	0	25
3	LG	T3 N2b M0	20	0	0	0	1	21
4	BoT	T2 N0 M0	20	0	0	0	1	20
5	BoT	T3 N2c M0	40	1	0	0	0	14
6	BoT	T2 N2c M0	30	0	0	0	1	18
7	OT	T1 N1 M0	50	1	1	1	0	14
8	FoM	T2 N2c M0	30	1	1	0	1	21
9	FoM	T1 N2b M0	20	0	0	0	0	18
10	OT	T2 N1 M0	40	1	1	0	0	16
11	MAR	T3 N0 M0	40	1	1	0	0	23
12	FoM	T3 N0 M0	20	0	0	0	1	20
13	FoM	T2 N2b M0	50	1	0	1	1	30
14	LG	T2 N0 M0	60	1	0	0	0	14
15	SSG	T3 N0 M0	30	0	0	0	0	15
16	OT	T1 N0 M0	30	0	0	0	0	15
17	OT	T3 N2b M0	30	0	0	0	1	21
18	FoM	T1 N0 M0	20	0	0	0	0	17
19	BM	T3 N2b M0	30	0	0	1	1	18
20	BoT	T1 N0 M0	20	0	0	0	0	28
21	FoM	T4a N2c M0	50	1	0	0	1	24

Abbreviations: OT—oral tongue, BM—buccal mucosa, LG—lower gingiva, BoT—base of tongue, FoM—floor of mouth, MAR—maxillary alveolar ridge, SSG—submandibular salivary gland, 0—absence of test feature, 1—presence of test feature.

**Table 2:** Measurements of standardized uptake value (SUV) values, tumor geometrical parameters and number of metastatic lymph nodes obtained from positron emission tomography/magnetic resonance (PET/MR) and computed tomography (CT), as well as biochemical parameters in pts (patients) with head and neck squamous cell carcinoma.

No of pts	SUVmax [g/mL]	SUVmean [g/mL]	Maximal Tumor Diameter CT [mm]	Maximal Tumor Diameter PET/MR [mm]	Tumor Volume PET/MR [cm^3^]	Tumor Volume CT [cm^3^]	No of mts Lymph Nodes in CT	No of mts Lymph Nodes in PET/MR	CRP [mg/L]	Glucose [mg/dL]
1	11.7	6.03	34	33	12.63	6.65	0	1	1.9	92
2	11.9	7.68	15	21	9.62	11.24	2≤	0	0.5	110
3	9.25	7.96	45	53	18.23	13.97	2≤	2≤	3.1	99
4	12.1	7.09	27	26	7.76	4.35	0	1	9.1	139
5	15.2	10.1	50	55	55.48	51.26	2≤	2≤	1.1	88
6	9.26	5.09	28	13	13.38	19.51	2≤	2≤	0.9	99
7	10.1	6.7	24	19	9.76	4.78	0	1	0.4	93
8	7.08	5.1	51	38	78.35	32.12	2≤	2≤	1.1	117
9	15.3	13.3	10	48	24.27	15.06	0	2≤	4.2	122
10	5.59	5.35	25	23	10.31	5.09	0	1	1.2	104
11	7.59	8.84	35	38	41.97	21.82	2≤	2≤	44.4	91
12	10	7.65	41	43	36.2	35.65	0	0	0.9	83
13	3.62	3.64	18	15	6.91	6.07	2≤	2≤	0.7	92
14	13.9	10.7	18	42	13.79	5.89	2≤	1	3.6	118
15	12.3	12.5	39	49	47.08	64.8	1	0	0.3	93
16	6.11	2.61	25	21	7.16	4.08	0	0	16	85
17	8.9	7.26	0	46	28.85	26.4	2≤	2≤	120.5	98
18	7.59	5.43	19	20	8.71	5.66	0	0	17.4	122
19	18.2	12.5	39	50	24.37	21.34	2≤	2≤	2.1	104
20	2.5	2.6	20	16	3.28	4.12	0	0	0.6	87
21	6.9	8.41	60	63	64.14	62.03	1	0	5.1	101

## References

[1] Burela N., Soni T., Patni N., Bhagat J., Kumar T., Natarajan T. (2017). A quantitative comparison of gross tumor volumes delineated on [18F]-FDG-PET/CT scan and contrast-enhanced computed tomography scan in locally advanced head and neck carcinoma treated with intensity modulated radiotherapy. Adv. Mod. Oncol. Res..

[2] Kim S.G., Friedman K., Patel S., Hagiwara M. (2016). Potential role of PET/MRI for imaging metastatic lymph nodes in head and neck cancer. Am. J. Roentgenol..

[3] Szyszko T.A., Cook G.J.R. (2018). PET/CT and PET/MRI in head and neck malignancy. Clin. Radiol..

[4] Goel R., Moore W., Sumer B., Khan S., Sher D., Subramaniam R.M. (2017). Clinical practice in PET/CT for the management of head and neck squamous cell cancer. Am. J. Roentgenol..

[5] Zheng E., Khariwala S.S. (2018). Do All Patients with Head and Neck Cancer Require a Positron Emission Tomography Scan at Diagnosis?. Laryngoscope.

[6] Leclerc M., Lartigau E., Lacornerie T., Daisne J.F., Kramar A., Grégoire V. (2015). Primary tumor delineation based on 18FDG PET for locally advanced head and neck cancer treated by chemo-radiotherapy. Radiother. Oncol..

[7] Ligtenberg H., Jager E.A., Caldas-Magalhaes J., Schakel T., Pameijer F.A., Kasperts N., Philippens M.E. (2017). Modality-specific target definition for laryngeal and hypopharyngeal cancer on FDG-PET, CT and MRI. Radiother. Oncol..

[8] Tsujikawa T., Narita N., Kanno M., Takabayashi T., Fujieda S., Okazawa H. (2018). Role of PET/MRI in oral cavity and oropharyngeal cancers based on the 8th edition of the AJCC cancer staging system: A pictorial essay. Ann. Nucl. Med..

[9] Differding S., Hanin F.X., Grégoire V. (2015). PET imaging biomarkers in head and neck cancer. Eur. J. Nucl. Med. Mol. Imaging.

[10] Huang S.H., Chien C.Y., Lin W.C., Fang F.M., Wang P.W., Lui C.C., Chang C.C. (2011). A comparative study of fused FDG PET/MRI, PET/CT, MRI, and CT imaging for assessing surrounding tissue invasion of advanced buccal squamous cell carcinoma. Clin. Nucl. Med..

[11] Loeffelbein D.J., Souvatzoglou M., Wankerl V., Dinges J., Ritschl L.M., Mücke T., Beer A.J. (2014). Diagnostic value of retrospective PET-MRI fusion in head-and-neck cancer. BMC Cancer.

[12] Mehanna H., Jones T.M., Gregoire V., Ang K.K. (2010). Oropharyngeal carcinoma related to human papillomavirus. BMJ Br. Med. J..

[13] Lydiatt W.M., Patel S.G., O’Sullivan B., Brandwein M.S., Ridge J.A., Migliacci J.C., Shah J.P. (2017). Head and neck cancers—Major changes in the American Joint Committee on cancer eighth edition cancer staging manual. CA Cancer J. Clin..

[14] O’Sullivan B., Huang S.H., Su J., Garden A.S., Sturgis E.M., Dahlstrom K., Adelstein D. (2016). Development and validation of a staging system for HPV-related oropharyngeal cancer by the International Collaboration on Oropharyngeal cancer Network for Staging (ICON-S): A multicentre cohort study. Lancet Oncol..

[15] Tahari A.K., Alluri K., Quon H., Koch W., Wahl R.L., Subramaniam R.M. (2014). FDG PET/CT imaging of Oropharyngeal SCC: Characteristics of HPV positive and negative tumors. Clin. Nucl. Med..

[16] Sharma S.J., Wittekindt C., Knuth J., Steiner D., Wuerdemann N., Laur M., Klussmann J.P. (2017). Intraindividual homogeneity of 18F-FDG PET/CT parameters in HPV-positive OPSCC. Oral Oncol..

[17] Nesteruk M., Lang S., Veit-Haibach P., Studer G., Stieb S., Glatz S., Guckenberger M. (2015). Tumor stage, tumor site and HPV dependent correlation of perfusion CT parameters and [18F]-FDG uptake in head and neck squamous cell carcinoma. Radiother. Oncol..

[18] Kim Y.I., Cheon G.J., Kang S.Y., Paeng J.C., Kang K.W., Lee D.S., Chung J.K. (2018). Prognostic value of simultaneous 18 F-FDG PET/MRI using a combination of metabolo-volumetric parameters and apparent diffusion coefficient in treated head and neck cancer. EJNMMI Res..

[19] Grégoire V., Ang K., Budach W., Grau C., Hamoir M., Langendijk J.A., Lee A., Le Q.T., Maingon P., Nutting C. (2014). Delineation of the neck node levels for head and neck tumors: A 2013 update. DAHANCA, EORTC, HKNPCSG, NCIC CTG, NCRI, RTOG, TROG consensus guidelines. Radiother. Oncol..

[20] Schaarschmidt B.M., Heusch P., Buchbender C., Ruhlmann M., Bergmann C., Ruhlmann V., Schlamann M., Antoch G., Forsting M., Wetter A. (2016). Locoregional tumour evaluation of squamous cell carcinoma in the head and neck area: A comparison between MRI, PET/CT and integrated PET/MRI. Eur. J. Nucl. Med. Mol. Imaging.

[21] Chan S.C., Yeh C.H., Yen T.C., Ng S.H., Chang J.T., Lin C.Y., Yen-Ming T., Fan K.H., Huang B.S., Hsu C.L. (2018). Clinical utility of simultaneous whole-body 18 F-FDG PET/MRI as a single-step imaging modality in the staging of primary nasopharyngeal carcinoma. Eur. J. Nucl. Med. Mol. Imaging.

[22] Sekine T., de Galiza Barbosa F., Kuhn F.P., Burger I.A., Stolzmann P., Huber G.F., Kollias S.S., von Schulthess G.K., Veit-Haibach P., Huellner M.W. (2017). PET+ MR versus PET/CT in the initial staging of head and neck cancer, using a trimodality PET/CT+ MR system. Clin. Imaging.

[23] De Bondt R.B., Nelemans P.J., Hofman P.A., Casselman J.W., Kremer B., van Engelshoven J.M., Beets-Tan R.G. (2007). Detection of lymph node metastases in head and neck cancer: A meta-analysis comparing US, USgFNAC, CT and MR imaging. Eur. J. Radiol..

[24] Altman D.G., Bland J.M. (1994). Statistics Notes: Diagnostic tests 2: Predictive values. BMJ.

[25] Torabi M., Aquino S.L., Harisinghani M.G. (2004). Current concepts in lymph node imaging. J. Nucl. Med..

[26] Platzek I., Beuthien-Baumann B., Schneider M., Gudziol V., Langner J., Schramm G., Laniado M., Kotzerke J., van den Hoff J. (2013). PET/MRI in head and neck cancer: Initial experience. Eur. J. Nucl. Med. Mol. Imaging.

[27] Geraldine B.E., Pyatigorskaya N., De Laroche R., Herve G., Zaslavsky C., Bertaux M., Giron A., Soret M., Bertolus C., Sahli-Amor M. (2017). Diagnostic performance of 18F-FDG PET/MR in head and neck malignancies. J. Nucl. Med..

[28] Grégoire V., Thorwarth D., Lee J. (2018). Molecular imaging-guided radiotherapy for the treatment of head-and-neck squamous cell carcinoma: Does it fulfill the promises?. Semin. Radiat. Oncol..

[29] Samołyk-Kogaczewska N., Sierko E., Zuzda K., Gugnacki P., Szumowski P., Mojsak M., Burzyńska-Śliwowska J., Wojtukiewicz M.Z., Szczecina K., Jurgilewicz D.H. (2019). PET/MRI-guided GTV delineation during radiotherapy planning in patients with squamous cell carcinoma of the tongue. Strahlenther. Onkol..

[30] Kocher M.R., Sharma A., Garrett-Mayer E., Ravenel J.G. (2018). Pretreatment 18F-Fluorodeoxyglucose Positron Emission Tomography Standardized Uptake Values and Tumor Size in Medically Inoperable Nonsmall Cell Lung Cancer Is Prognostic of Overall 2-Year Survival after Stereotactic Body Radiation Therapy. J. Comput. Assist. Tomo..

[31] Rutkowski T. (2014). The role of tumor volume in radiotherapy of patients with head and neck cancer. Radiat. Oncol..

[32] Paul A.G., Heilbrun L.K., Smith D.W., Miller S.R. (2018). Association of 18F-FDG-PET SUV and Tumor Size in Cervical Cancer. Int. J. Radiat. Oncol..

[33] Deng S.M., Zhang W., Zhang B., Chen Y.Y., Li J.H., Wu Y.W. (2015). Correlation between the uptake of 18F-fluorodeoxyglucose (18F-FDG) and the expression of proliferation-associated antigen Ki-67 in cancer patients: A meta-analysis. PLoS ONE.

[34] Huang H., Xiao F., Han X., Zhong L., Zhong H., Xu L., Zhu J., Ni B., Liu J., Fang Y. (2016). Correlation of pretreatment 18F-FDG uptake with clinicopathological factors and prognosis in patients with newly diagnosed diffuse large B-cell lymphoma. Nucl. Med. Commun..

[35] Surov A., Meyer H.J., Wienke A. (2019). Associations between PET parameters and expression of Ki-67 in breast cancer. Transl. Oncol..

[36] Ucar E., Yalcin H., Kavvasoglu G.H., Ilhan G. (2018). Correlations between the maximum standard uptake value of positron emission tomography/computed tomography and laboratory parameters before and after treatment in patients with lymphoma. Chin. Med. J..

[37] Eskian M., Alavi A., Khorasanizadeh M., Viglianti B.L., Jacobsson H. (2019). Effect of blood glucose level on standardized uptake value (SUV) in 18 F-FDG PET-scan: A systematic review and meta-analysis of 20, 807 individual SUV measurements. Eur. J. Nucl. Med. Mol. Imaging.

[38] Higashi T., Saga T., Nakamoto Y., Ishimori T., Fujimoto K., Doi R., Imamura M., Konishi J. (2003). Diagnosis of pancreatic cancer using fluorine-18 fluorodeoxyglucose positron emission tomography (fdg pet)—Usefulness and limitations in “clinical reality”. Ann. Nucl. Med..

[39] Westerterp M., Sloof G.W., Hoekstra O.S., ten Kate F.J., Meijer G.A., Reitsma J.B., Boellaard R., van Lanschot J.J., Molthoff C.F. (2008). 18 FDG uptake in oesophageal adenocarcinoma: Linking biology and outcome. J. Cancer Res. Clin..

[40] Fleming J.C., Woo J., Moutasim K., Mellone M., Frampton S.J., Mead A., Woelk C.H. (2019). HPV, tumour metabolism and novel target identification in head and neck squamous cell carcinoma. Br. J. Cancer.

[41] Han M., Lee S.J., Lee D., Kim S.Y., Choi J.W. (2018). Correlation of human papilloma virus status with quantitative perfusion/diffusion/metabolic imaging parameters in the oral cavity and oropharyngeal squamous cell carcinoma: Comparison of primary tumour sites and metastatic lymph nodes. Clin. Radiol..

[42] Noij D.P., Martens R.M., Zwezerijnen B., Koopman T., de Bree R., Hoekstra O.S., Castelijns J.A. (2018). Diagnostic value of diffusion-weighted imaging and 18F-FDG-PET/CT for the detection of unknown primary head and neck cancer in patients presenting with cervical metastasis. Eur. J. Radiol..

[43] Schouten C.S., Hakim S., Boellaard R., Bloemena E., Doornaert P.A., Witte B.I., de Bree R. (2016). Interaction of quantitative 18F-FDG-PET-CT imaging parameters and human papillomavirus status in oropharyngeal squamous cell carcinoma. Head Neck.

[44] Surov A., Meyer H.J., Höhn A.K., Winter K., Sabri O., Purz S. (2019). Associations between [18 F] FDG-PET and complex histopathological parameters including tumor cell count and expression of KI 67, EGFR, VEGF, HIF-1α, and p53 in head and neck squamous cell carcinoma. Mol. Imaging Biol..

[45] Ahmedova A., Ozkaya K., Tambas M., Gezer U., Ozgur E., Sahin D., Altun M. (2017). Is there a correlation between serum Epstein-Barr virus DNA level and tumor metabolic activity, TNM staging and tumor load in nasopharyngeal cancer patients?. J. Clin. Oncol..

[46] Alessi A., Lorenzoni A., Cavallo A., Padovano B., Iacovelli N.A., Bossi P., Alfieri S., Serafini G., Colombo C.B., Cicchetti A. (2019). Role of pretreatment 18F-FDG PET/CT parameters in predicting outcome of non-endemic EBV DNA-related nasopharyngeal cancer (NPC) patients treated with IMRT and chemotherapy. La Radiologia Medica.

[47] Rischin D., Young R.J., Fisher R., Fox S.B., Le Q.T., Peters L.J., Solomon B., Choi J., O’Sullivan B., Kenny L.M. (2010). Prognostic significance of p16INK4A and human papillomavirus in patients with oropharyngeal cancer treated on TROG 02.02 phase III trial. J. Clin. Oncol..

[48] Chen Q.Y., Guo S.Y., Tang L.Q., Lu T.Y., Chen B.L., Zhong Q.Y., Liu L.T. (2018). Combination of tumor volume and Epstein-Barr virus DNA improved prognostic stratification of stage II nasopharyngeal carcinoma in the intensity modulated radiotherapy era: A large-scale cohort study. Cancer Res. Treat..

[49] Lu L., Li J., Zhao C., Xue W., Han F., Tao T., Lu T. (2016). Prognostic efficacy of combining tumor volume with Epstein-Barr virus DNA in patients treated with intensity-modulated radiotherapy for nasopharyngeal carcinoma. Oral. Oncol..

[50] Peng L., Yang Y., Guo R., Mao Y.P., Xu C., Chen Y.P., Tang L.L. (2018). Relationship between pretreatment concentration of plasma Epstein-Barr virus DNA and tumor burden in nasopharyngeal carcinoma: An updated interpretation. Cancer Med..

[51] Zhong Q., Xiao Z.Y., Wu R.R. (2010). The correlation of EBV in plasma and primary tumor volume of NPC. J. Gannan Med Univ..

[52] Gletsou E., Papadas T.A., Baliou E., Tsiambas E., Ragos V., Armata I.E., Fotiades P.P. (2018). HPV infection in oropharyngeal squamous cell carcinomas: Correlation with tumor size. J. BUON.

[53] Liang S.B., Deng Y.M., Zhang N., Lu R.L., Zhao H., Chen H.Y., Chen Y. (2013). Prognostic significance of maximum primary tumor diameter in nasopharyngeal carcinoma. BMC Cancer.

[54] Kågedal Å., Rydberg Millrud C., Häyry V., Kumlien Georén S., Lidegran M., Munck-Wikland E., Cardell L.O. (2018). Oropharyngeal squamous cell carcinoma induces an innate systemic inflammation, affected by the size of the tumour and the lymph node spread. Clin. Otolaryngol..

[55] De Bree R., Takes R.P., Castelijns J.A., Medina J.E., Stoeckli S.J., Mancuso A.A., Hunt J.L., Rodrigo J.P., Triantafyllou A., Teymoortash A. (2015). Advances in diagnostic modalities to detect occult lymph node metastases in head and neck squamous cell carcinoma. Head Neck.

[56] Chen C., Chen Z., Cao H., Yan J., Wang Z., Le H., Weng J., Zhang Y. (2018). A retrospective clinicopathological study of lung adenocarcinoma: Total tumor size can predict subtypes and lymph node involvement. Clin. Imaging.

[57] Miller T.R., Grigsby P.W. (2002). Measurement of tumor volume by PET to evaluate prognosis in patients with advanced cervical cancer treated by radiation therapy. Int. J. Radiat. Oncol..

[58] Ouyang M.L., Xia H.W., Xu M.M., Lin J., Wang L.L., Zheng X.W., Tang K. (2019). Prediction of occult lymph node metastasis using SUV, volumetric parameters and intratumoral heterogeneity of the primary tumor in T1-2N0M0 lung cancer patients staged by PET/CT. Ann. Nucl. Med..

[59] Kim S.J., Pak K., Chang S. (2016). Determination of regional lymph node status using 18F-FDG PET/CT parameters in oesophageal cancer patients: Comparison of SUV, volumetric parameters and intratumoral heterogeneity. Br. J. Radiol..

[60] Raman A., Sen N., Ritz E., Fidler M.J., Revenaugh P., Stenson K., Al-khudari S. (2019). Heterogeneity in the clinical presentation, diagnosis, and treatment initiation of p16-positive oropharyngeal cancer. Am. J. Otol..

[61] Mirzamani N., Salehian P., Farhadi M., Amin Tehran E. (2007). Detection of EBV and HPV in nasopharyngeal carcinoma by in situ hybridization. Exp. Mol. Pathol..

[62] Niedobitek G. (2000). Epstein-Barr virus infection in the pathogenesis of nasopharyngeal carcinoma. Mol. Pathol..

[63] Higuchi T., Fujimoto Y., Ozawa H., Bun A., Fukui R., Miyagawa Y., Imamura M., Kitajima K., Yamakado K., Miyoshi Y. (2019). Significance of Metabolic Tumor Volume at Baseline and Reduction of Mean Standardized Uptake Value in 18 F-FDG-PET/CT Imaging for Predicting Pathological Complete Response in Breast Cancers Treated with Preoperative Chemotherapy. Ann. Surg. Oncol..

[64] Śnietura M., Jaworska M., Pigłowski W., Goraj-Zając A., Woźniak G., Lange D. (2010). High-risk HPV DNA status and p16 (INK4a) expression as prognostic markers in patients with squamous cell cancer of oral cavity and oropharynx. Pol. J. Pathol..

[65] Begum S., Gillison M.L., Ansari-Lari M.A., Shah K., Westra W.H. (2003). Detection of human papillomavirus in cervical lymph nodes: A highly effective strategy for localizing site of tumor origin. Clin. Cancer Res..

[66] Guzińska-Ustymowicz K., Pryczynicz A., Kemona A., Czyżewska J. (2009). Correlation between proliferation markers: PCNA, Ki-67, MCM-2 and antiapoptotic protein Bcl-2 in colorectal cancer. Anticancer Res..

[67] Altman D.G., Bland J.M. (1994). Diagnostic tests. 1: Sensitivity and specificity. BMJ Br. Med. J..

